# Self-Association of Purified Reconstituted ER Luminal Spacer Climp63

**DOI:** 10.3389/fcell.2020.00500

**Published:** 2020-06-16

**Authors:** Jinghua Zhao, Junjie Hu

**Affiliations:** ^1^Department of Genetics and Cell Biology, College of Life Sciences, Nankai University, Tianjin, China; ^2^National Laboratory of Biomacromolecules, CAS Center for Excellence in Biomacromolecules, Institute of Biophysics, Chinese Academy of Sciences, Beijing, China

**Keywords:** endoplasmic reticulum, sheet biogenesis, membrane tethering, homotypic interactions, reconstitution, Climp63

## Abstract

Membranes of the endoplasmic reticulum (ER) are shaped into cisternal sheets and cylindrical tubules. How ER sheets are generated and maintained is not clear. ER membrane protein Climp63 is enriched in sheets and routinely used as a marker of this structure. The luminal domain (LD) of Climp63 is predicted to be highly helical, and it may form bridges between parallel membranes, regulating the abundance and width of ER sheets. Here, we purified the LD and full-length (FL) Climp63 to analyze their homotypic interactions. The N-terminal tagged LD formed low-order oligomers in solution, but was extremely aggregation-prone when the GST tag was removed. Purified FL Climp63 formed detectable but moderate interactions with both the FL protein and the LD. When Climp63 was reconstituted into proteoliposomes with its LD facing out, the homotypic interactions were retained and could be competed by soluble LD, though vesicle clustering was not observed. These results demonstrate a direct self-association of Climp63, supporting its role as an ER luminal spacer.

## Introduction

The endoplasmic reticulum (ER) is composed by two interconnected morphological domains: tubules and sheets (Baumann and Walz, 2001; Shibata et al., 2006). Though the morphogenesis of the tubular ER network has been studied extensively (Shibata et al., 2009; Hu et al., 2011; Lin et al., 2012), little is known about how sheets are formed. ER sheets are cisternal structures bounded by two flattened parallel membranes, with a width of ∼30 nm in yeast and ∼50 nm in mammalian cells. Most sheets are decorated by translating ribosomes (Savitz and Meyer, 1990; Puhka et al., 2007), termed the rough ER, which links ER sheets to protein synthesis, a key function of the ER.

**FIGURE 1:**
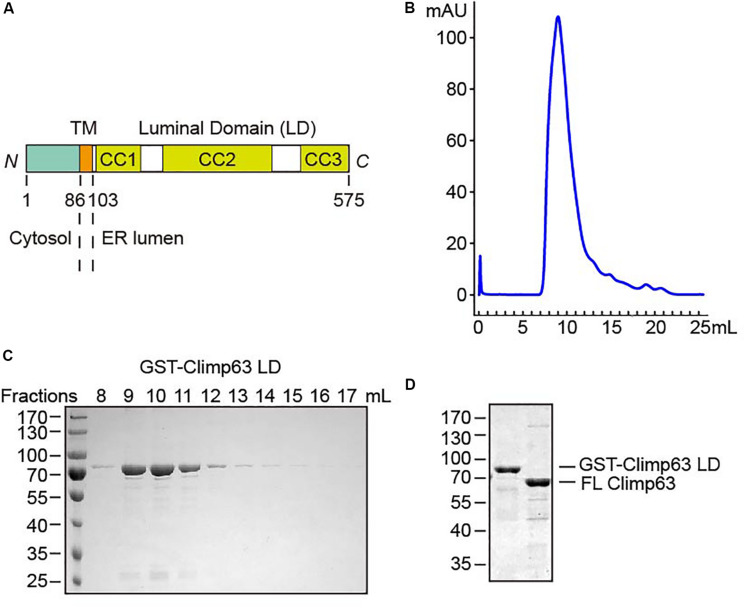
Purification of Climp63. **(A)** Schematic diagram of *M. musculus* Climp63. The amino acids are numbered. TM, transmembrane; CC, coiled coil. **(B)** GST-Climp63 LD was purified through gel filtration analysis. **(C)** After gel filtration, the fractions were analyzed by SDS-PAGE and Coomassie blue staining. **(D)** GST-Climp63 LD and FL Climp63 were purified and the samples were analyzed by SDS-PAGE and Coomassie blue staining.

Investigation of professional secretory cells, such as pancreatic cells and plasma cells that contain massive ER sheets, sheds light on key regulators of sheet formation (Shibata et al., 2010). Three ER membrane proteins, cytoskeleton-linking membrane protein 63 (Climp63), p180, and kinectin, have been identified as sheet-enriched proteins that determine ER sheet formation. Climp63 is proposed to serve as a luminal ER spacer by forming luminal bridges. p180 and kinectin, both of which potentially contain extensive cytosolic coiled coil domains, are thought to flatten ER membranes using these coiled coils. In addition, the presence of polysomes on ER membranes likely promotes sheet formation (Shibata et al., 2010). Finally, the shaping of ER sheets may be facilitated by tubule-forming proteins, which also localize to the edge of sheets and stabilize the high curvature there (Shibata et al., 2010). Among sheet-forming proteins, Climp63, which is also referred to as cytoskeleton associated protein 4 (CKAP4), is the most commonly used sheet marker in the ER. It rarely localizes to ER tubules (Gao et al., 2019; Schroeder et al., 2019) and is not observed in the nuclear envelope (Shibata et al., 2010). Climp63 is a 63 kDa type II transmembrane protein with a relatively short N-terminus (NT, 85 aa) and a long C-terminal luminal domain (LD, 472 aa; Figure 1A). Climp63 interacts with microtubules, as indicated by its name, through a region in the NT. The cytoskeleton linkage can be reversed by phosphorylation in the proximal region (Vedrenne et al., 2005) and potentially regulates translocon mobility (Nikonov et al., 2007) and ER positioning (Cui-Wang et al., 2012; Farías et al., 2019). Palmitoylation was also detected in the NT (Planey et al., 2009), but the physiological role is not entirely clear. Interestingly, Climp63 is also implicated in several signaling events (Nisha Gupta et al., 2006; Sandoz and van der Goot, 2015; Chavda et al., 2017), with some mechanisms requiring plasma membrane localization.

Importantly, the LD of Climp63 is expected to convey the most critical function, i.e., sheet morphogenesis. Depletion or deletion of Climp63 in cultured cells causes an ∼50% decrease in the luminal width of the ER (Shibata et al., 2010), and reintroduction of Climp63 with engineered LDs of different lengths results in ER sheets of corresponding width (Shen et al., 2019). The Climp63 LD is predicted to be mostly coiled coils (CCs), and homotypic zippering of these helices is naturally thought to be the basis for bridging the apposing lumen-forming membranes. However, purified LD was only analyzed in a denatured condition (Klopfenstein et al., 2001), and direct evidence of the existence of homotypic interactions is not available.

Here, we purified full-length (FL) Climp63 and the LD of Climp63 for biochemical analysis. We confirmed the helical nature of the LD and the self-association of Climp63 in both soluble and reconstituted forms, though the interactions were weaker than expected.

## Results

### Purification of the Climp63 LD

To test the direct homotypic interaction of Climp63, we expressed GST-tagged mouse Climp63 LD (residues 104–575, Supplementary Figure S1) in *Escherichia coli*. When affinity-purified GST-Climp63 LD (theoretical molecular weight: 80.3 kDa) was subject to size exclusion chromatography, the protein was eluted as a relatively broad range (∼8–12 ml, Figures 1B,C), the peak of which correlates to a calibrated molecular weight of ∼1000 kDa. On the same column, a monomeric protein of 80 kDa would elute at ∼14 ml. These results suggest that the LD likely forms oligomers and/or adopts an extended configuration that shortens its retention time in the column compared to other globular proteins.

To avoid an influence by the GST tag, which is known to form weak dimers in solution (Sacchetta et al., 1993), on the oligomeric state of the LD, we attempted to remove the tag using protease 3C. As expected, the GST was efficiently separated from the LD by overnight cleavage on glutathione beads (Supplementary Figure S2A). The released LD severely precipitated, whereas uncleaved protein was stable in solution when eluted from the beads. These results indicate that isolated LD is aggregation-prone, likely due to a sticky surface that can be protected by the GST.

### Tests of Self-Association Using Purified Climp63

**FIGURE 2:**
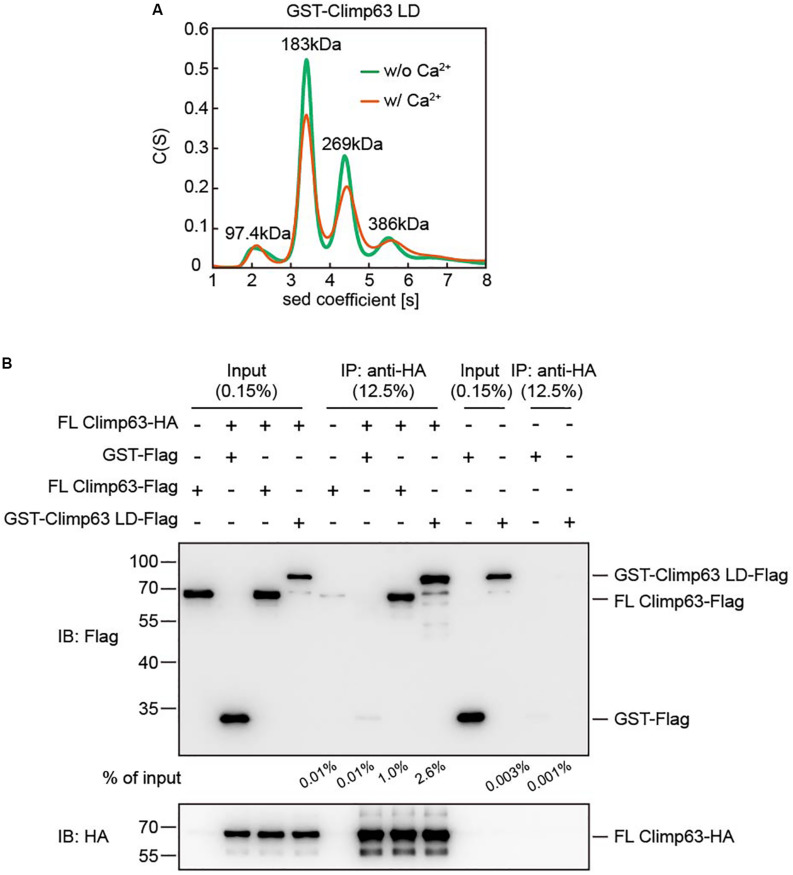
Interactions between purified Climp63. **(A)** The size of GST-Climp63 LD (theoretical molecular weight: 80.3 kDa) was determined at 12.5 μM by analytical ultracentrifugation (AUC) with or without 2 mM CaCl_2_. The estimated molecular masses are given above the peaks (in kilodaltons, kDa). **(B)** The interaction between purified FL Climp63-HA and FL Climp63-Flag was determined by co-immunoprecipitation. 5 μg of purified Climp63-HA (0.16 μM) was immunoprecipitated by anti-HA-agarose and then incubated with 10 μg of Flag-tagged protein, including FL Climp63-Flag (0.32 μM), GST-Climp63 LD-Flag (0.25 μM), or GST-Flag (0.8 μM), at 4°C for 1.5 h. The samples were analyzed by immunoblotting using the indicated antibodies. 0.15% of the input and 12.5% of the precipitate was loaded. The relative levels of co-precipitated Flag-proteins were quantified using the Gel-Pro analyzer software and normalized to the levels of input. Data are representative of three biological repeats.

Next, we tested the oligomeric state of the GST-Climp63 LD using analytical ultra-centrifugation (AUC). When 12.5 μM protein was loaded, the GST-Climp63 LD was mostly dimer (∼44%), but we also observed monomer (7.5%), trimer (∼28%) and tetramer (∼13%) species (Figure 2A). Because GST forms dimers (Supplementary Figure S3A), but not trimers (Sacchetta et al., 1993), these results suggest that Climp63 LD undergoes moderate self-association in addition to that by the GST. Furthermore, the oligomerization state was not affected in the presence of 2 mM Ca^2+^ (Figure 2A), which mimics the conditions with the ER lumen. The results also suggest that the high average molecular weight predicted by gel filtration analysis when similar protein concentrations were used is caused at least partly by an irregular shape of the protein, such as a rod, as proposed previously (Klopfenstein et al., 2001).

To further confirm the LD-mediated homotypic interactions, we performed pull-down assays using purified proteins. Because the GST-tag cannot be removed and its presence may cause background association, we expressed and purified GST-tagged FL mouse Climp63 (Figure 1D). Unlike the LD, the N-terminal GST could be cleaved efficiently without compromising the stability of the protein (Supplementary Figure S2B). We then engineered and purified FL Climp63 with either a C-terminal HA tag or Flag tag, the GST-Climp63 LD with a C-terminal Flag tag, and GST-Flag. FL Climp63-HA was attached to anti-HA antibody-conjugated agarose, incubated with individual Flag-tagged proteins, and the precipitates analyzed by SDS-PAGE and immunoblotting using anti-HA and anti-Flag antibodies. Both FL Climp63-Flag and GST-Climp63 LD-Flag were detected in the precipitates, but very little GST-Flag co-precipitated with FL Climp63-HA (Figure 2B). Notably, ∼0.5–1 μM total protein was used in these assays, and FL Climp63-HA was only able to pull down ∼1.0% of the FL Climp63-Flag or ∼2.6% of the LD that was supplied. When FL Climp63-HA was omitted in the assay, FL Climp63-Flag had a marginal attachment to the anti-HA agarose (Figure 2B). These results confirm a detectable, but relatively weak, homotypic interaction with Climp63.

The LD of Climp63 is predicted to be entirely α-helical with three potential CC regions (Supplementary Figure S1). To test whether deletion of the CC domains affects homotypic interactions, we preformed pull-down assays using deletion mutants in the context of FL Climp63 with an N-terminal Strep tag (Supplementary Figure S3B). As expected, Strep-FL Climp63 was able to co-sediment with FL Climp63-HA. Decreased binding was observed when CC2, the longest CC, was deleted, and the lack of both CC1 and CC2 caused a further reduction in self-association (Supplementary Figure S3C). These results suggest that redundant binding sites exist in the LD, and Climp63 self-association is sensitive to CC deletions.

### Reconstitution of FL Climp63

**FIGURE 3:**
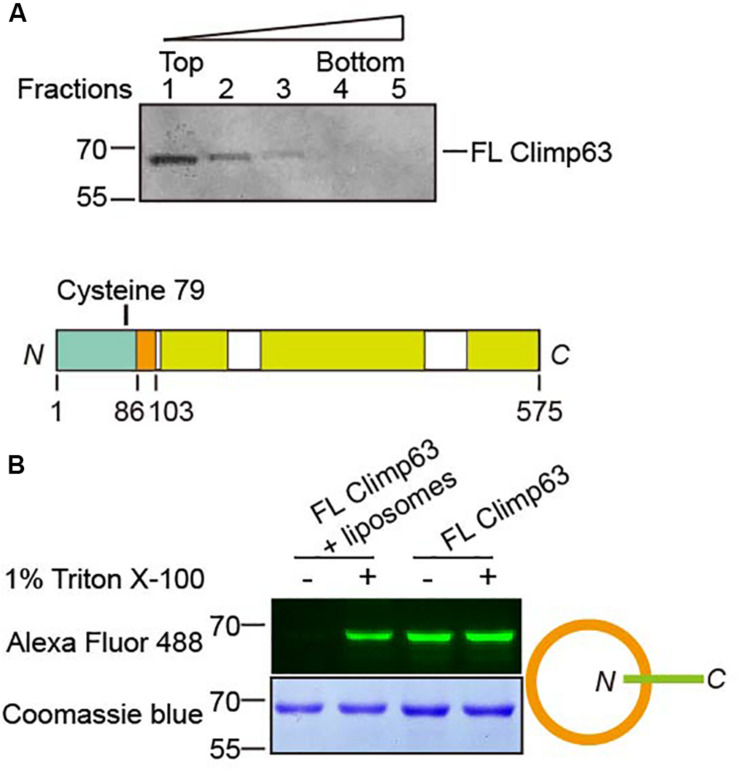
Reconstitution of Climp63. **(A)** FL Climp63 was reconstituted into proteoliposomes followed by flotation analysis. Fractions were analyzed by SDS-PAGE and Coomassie blue staining. **(B)** The top panel shows a single cysteine in *M. musculus* Climp63. Purified and reconstituted FL Climp63 were labeled by a 10-fold molar excess of Alexa Fluor 488 C5 maleimide dye, with or without 1% Triton X-100, overnight at 4°C. The reactions were stopped by 2 mM BME. Samples were separated by SDA-PAGE and analyzed by fluorescent imaging (top) and Coomassie blue staining (bottom). The orientation of the reconstitution is indicated on the right.

We also tested whether Climp63 alters homotypic interactions in a membrane-bound setting. To achieve this goal, we performed directional reconstitution of the purified FL Climp63. Climp63 was solubilized by Fos-Choline-12 and subsequently exchanged into Triton X-100. In the reconstitution mixture, when the chosen detergent, including Triton X-100, is sufficient to be incorporated into pre-formed liposomes, but not enough to solubilize them, removal of the detergent could possibly cause directional insertion of membrane proteins into lipid bilayers (Rigaud and Levy, 2003). Using this method, we reconstituted FL Climp63 into proteoliposomes with bio-bead-aided detergent removal. Flotation analysis of the reconstituted sample indicated that Climp63 was successfully incorporated into membrane vesicles (Figure 3A). Climp63 contains one cysteine residue in the NT (C79, Figure 3B); accessibility of C79 by maleimide-linked Alexa Fluor 488 was used to probe the topology of reconstituted Climp63. Purified FL Climp63 was labeled efficiently. When Climp63-containing proteoliposomes were intact, no labeling was detected. Fluorescent labeling was resumed when the same sample was solubilized by 1% Triton X-100 (Figure 3C). We also mutated C79 to Ala and reintroduced a Cys at S124 in the LD domain. As expected, the reconstituted double mutant was readily labeled when the proteoliposomes were intact (Supplementary Figure S4A). Taken together, these results indicate that Climp63 was reconstituted into membranes with the LD/CT facing out.

### Tests of Self-Association Using Reconstituted Climp63

**FIGURE 4:**
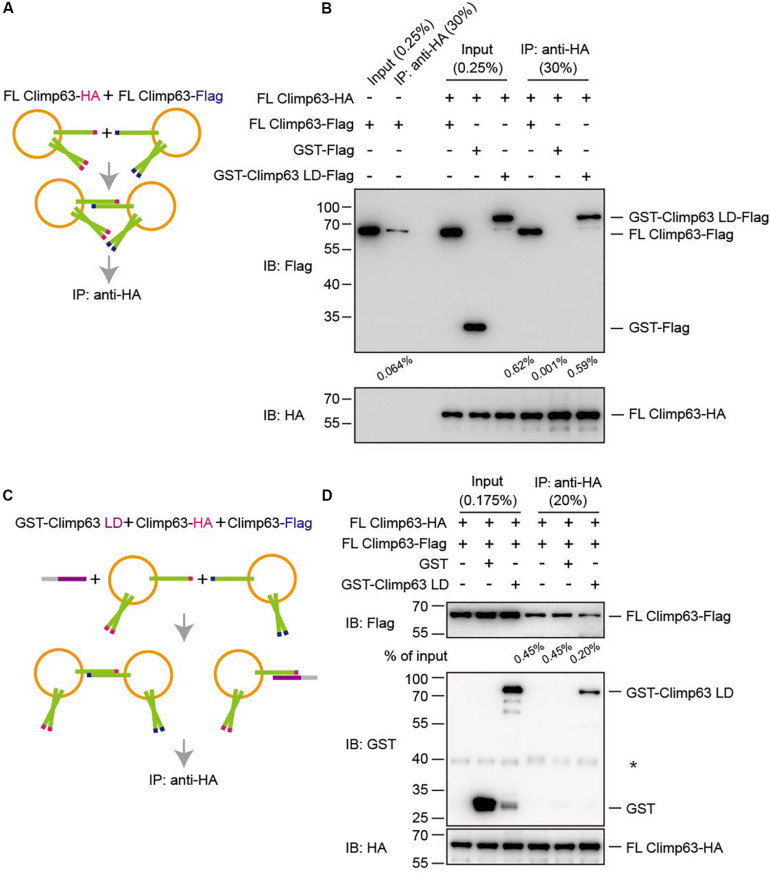
Interactions between reconstituted Climp63. **(A)** Schematic diagram of the pull-down process shown in **(B)**. **(B)** FL Climp63-HA and FL Climp63-Flag were individually reconstituted into proteoliposomes and subjected to flotation. The top fractions (50 μl of 250 μl) were used for the pull-down assays. FL Climp63-HA-containing vesicles (∼0.75 μg protein, 0.03 μM) were mixed with the indicated Flag-containing samples, including FL Climp63-Flag-containing vesicles (∼1.5 μg protein, 0.06 μM), GST-Climp63 LD-Flag (1.5 μg, 0.05 μM), and GST-Flag (1.5 μg, 0.15 μM), and precipitated by anti-HA agarose. The samples were analyzed by immunoblotting using the indicated antibodies. 0.25% of the input and 30% of the precipitate was loaded. The relative levels of co-precipitated Flag-proteins were quantified using the Gel-Pro analyzer software and normalized to the levels of input. Data are representative of three biological repeats. **(C)** Schematic diagram of the pull-down process shown in **(D)**. **(D)** FL Climp63-HA and FL Climp63-Flag were individually reconstituted into proteoliposomes. FL Climp63-HA-containing vesicles (∼0.1 μM) were mixed with FL Climp63-Flag-containing vesicles (∼0.15 μM). GST-Climp63 LD (0.9 μM) was added for competition and GST (3 μM) was used as a control. The samples were analyzed by immunoblotting using the indicated antibodies. 0.175% of the input and 20% of the precipitate was loaded. The relative levels of co-precipitated Flag-proteins were quantified using the Gel-Pro analyzer software and normalized to the levels of input. The asterisk (^∗^) indicates a non-specific band. Data are representative of three biological repeats.

Finally, Climp63-mediated interactions were measured in a proteoliposome-based pull-down assay. Vesicles containing either FL Climp63-HA or FL Climp63-Flag were isolated by collecting the top fraction in the flotation assay (Supplementary Figure S4B). Cryo-EM showed that proteoliposomes remained intact upon flotation, but no obvious tethering between vesicles was observed (Supplementary Figure S4C). These vesicles were then mixed and incubated with HA-agarose to pull-down FL Climp63-HA-containing vesicles (Figure 4A). A very low concentration of detergent (0.01% Triton X-100) was maintained, not to break vesicles, but to ensure binding fidelity in the assay. Consistently, HA-positive vesicles were able to co-sediment some Flag-positive vesicles (Figure 4B). In addition, when GST-Climp63 LD-Flag was incubated with FL Climp63-HA-containing vesicles, the LD was also detected in the HA-positive precipitates (Figure 4B). However, when the LD was present in 6-fold molar excess (Figure 4C), the interactions between HA-positive vesicles and Flag-positive vesicles were reduced (Figure 4D). Collectively, these results confirm that membrane-bound Climp63 is able to self-associate through its LD domain.

## Discussion

Our results provide definitive evidence that Climp63 may act as an ER luminal spacer by forming homotypic interactions through the ER LD. The self-association can reach at least tetramers in solution. We confirmed the interactions using the LD alone, between the LD and FL Climp63, and between differentially tagged FL Climp63 in the presence or absence of detergents. The assembly is likely specific, because purified proteins used in the binding assays did not form uncontrollable aggregations and the assembly can be competed by the soluble LD and down-regulated by CC deletion.

Surprisingly, the measured affinity of the interaction was weaker than expected given that the LD is predicted to form extensive CCs. Marginal amounts of the LD or FL Climp63 were pulled down by HA-agarose-precipitated FL Climp63. Even when the protein concentration was 10-fold higher, the degree of oligomer formation by GST-Climp63 LD was maintained at low levels. These findings suggest that, although Climp63-mediated luminal bridges exist, they are less likely to be rigid and stable. Our results predict a self-association affinity of Climp63 that is weaker than micromolar. In contrast, a similar luminal bridge formed by KASH protein in the outer nuclear membranes and SUN protein in the inner nuclear membranes was reported to be ∼45 nM (Wang et al., 2012). When tethering factors, such as Climp63, are densely placed in apposed 2D faces, weak interactions may be combined to a reasonable level. Similar cases include Golgi stacking factors, the GRASP proteins. They are purified as monomers, but the crystal structures have revealed multiple assembly interfaces that are of physiological relevance in cells (Feng et al., 2013). Alternatively, the flexible bridges allow dynamic regulation of the ER luminal space.

We noticed that isolated LD exhibited severe aggregation when the GST tag was removed. These findings suggest possible strong *cis* interactions, i.e., self-association of Climp63 in the same side of the membrane, which might undermine *trans* interactions that were tested here and indeed needed as a luminal spacer. The width of ER sheets in mammalian cells is ∼50 nm in average. However, the previously purified Climp63 LD (in partial denatured conditions) was seen as 90-nm rods by EM, which less likely represents a functional spacer (Klopfenstein et al., 2001). If the LD forms one single straight helix, it would be estimated as 70 nm in length. Certain winding of the helical domain is therefore expected for a fit in 50 nm space.

Our findings imply that the Climp63 LD is most likely sticky and can bind to many other proteins in the ER lumen. The LD has been reported to bind to dicer (Pepin et al., 2012), integrin (Osugi et al., 2019), VE-cadherin (Lyu et al., 2019), and DKK1 (Kimura et al., 2016). Interestingly, Climp63 was recently shown to engage calumenin, a soluble ER chaperone (Shen et al., 2019), highlighting potential docking sites for luminal chaperones. Similarly, Climp63 was found to regulate the nanodomain distribution of ER-resident proteins (Gao et al., 2019). These observations suggest that Climp63 has functions beyond a ER luminal spacer, possibly linking to its ER sheet enrichment.

## Materials and Methods

### Protein Expression and Purification

Full-length Climp63 (*Mus musculus*), CC-deleted mutants, and Climp63 LD (residues 104–575) were cloned into the pGEX6P-1 vector with an N-terminal GST tag. All constructs were transformed into bacterial strain BL21 (DE3) and cultures grown in Luria-Bertani media at 37°C to an OD600 of 0.8. Protein expression was induced by the addition of 0.35 mM IPTG for 24 h at 16°C. Cells were harvested, resuspended in lysis buffer (500 mM NaCl, 25 mM HEPES [pH 7.4], 10% glycerol), and lysed by ultrasonication. The lysate was centrifuged at 40,000 rpm for 1 h. For FL Climp63, the pellet was resuspended in lysis buffer containing 1% Fos-choline-12 and the insoluble components cleared by centrifugation. The recombinant protein was isolated by glutathione Sepharose (GE Healthcare), washed with lysis buffer containing 0.1% Triton X-100, and eluted by cleavage of the GST tag, where 180 μg protease 3C was incubated with the resuspended beads overnight at 4°C. For the GST-Climp63 LD, the supernatant was incubated with glutathione Sepharose after centrifugation. Sepharose was washed with lysis buffer (500 mM NaCl, 25 mM HEPES [pH 7.4]) and then eluted with buffer containing 10 mM glutathione. The protein was further purified by gel filtration chromatography (Superdex-200 Increase 10/300 GL; GE Healthcare) in 25 mM HEPES (pH7.4) and 500 mM NaCl with a flowrate of 0.5 mL/min at 4°C.

### AUC

Purified 1 mg/mL GST-Climp63 LD (12.5 μM) and GST (40 μM) were used for AUC in a buffer containing 25 mM HEPES (pH7.4) and 500 mM NaCl. Sedimentation velocity experiments were performed at 4°C in Optima AUC (Beckman Coulter). All absorbance data at 280 nm were collected at 42,000 rpm in a rotor (An-50 Ti; Beckman Coulter) and analyzed by the program SEDFIT in terms of a continuous c(s) distribution.

### Reconstitution of FL Climp63

POPC (1-palmitoyl-2-oleoyl-sn-glycero-3-phosphocholine), DOPS (1,2-dioleoyl-sn-glycero- 3-phospho-L-serine) and Rhod-PE [1,2-dioleoyl-sn-glycero-3-phosphoethanolamine-N-(lissamine rhodamine B sulfonyl)] were purchased from Avanti Polar Lipids. Lipid mixes (POPC:DOPS:Rhod-PE, 83.5:15:1.5 molar ratio) were dried to a film, hydrated with buffer (500 mM NaCl, 25 mM HEPES [pH 7.4], 10% glycerol), and extruded through polycarbonate filters with a pore size of 100 nm. FL Climp63 in 0.1% Triton X-100 was mixed with preformed liposomes (protein:liposome, 1:1000 molar ratio) at an effective detergent to lipid ratio of ∼1. Protein and lipid were allowed to mix for 2 h at 4°C. The detergent was removed by adding Bio-Beads SM-2 adsorbent beads (Bio-Rad). The flotation of proteoliposomes was performed on a sucrose gradient to determine the reconstitution efficiency. Proteoliposomes (30 μl) were mixed with 100 μl of 1.9 M sucrose and overlaid with 100 μl of 1.25 M sucrose and 20 μl of lysis buffer. After centrifugation at 174,000 *g* for 75 min at 4°C in a rotor (TLS- 55; Beckman Coulter), the gradient was fractionated into five fractions and analyzed by SDS-PAGE and Coomassie blue staining.

### Protein Labeling

Purified FL Climp63 and proteoliposomes were labeled with 10-fold molar excess of Alexa Fluor 488 C5 maleimide dye (Life Technologies) in the absence or presence of 1% Triton X-100 overnight at 4°C. The samples were separated by SDA-PAGE and analyzed by fluorescent imaging and Coomassie blue.

### Immunoprecipitation

For immunoprecipitation of purified protein, 5 μg of FL Climp63-HA was immunoprecipitated by anti-HA-agarose at 4°C for 2 h. The agarose was washed twice with lysis buffer containing 0.1% digitonin and then incubated with 10 μg of Flag-tagged proteins in lysis buffer containing 1% digitonin at 4°C for 1.5 h. The agarose was washed four times with lysis buffer containing 0.1% digitonin. For immunoprecipitation of proteoliposomes, FL Climp63-HA and FL Climp63-Flag were reconstituted into liposomes and subjected to flotation. The top fractions were mixed in lysis buffer containing 0.0125% Triton X-100 at 4°C for 2 h. The mixture was then precipitated by anti-HA agarose at 4°C for 1.5 h. The agarose was washed four times with lysis buffer. Precipitated proteins were eluted with 2 × SDS-PAGE sample buffer and detected by Western blot. Band densities were quantified using Gel-Pro Analyzer version 4.0 (Media Cybernetics).

### Cryo-Electron Microscopy

Full-length Climp63 was reconstituted into liposomes and subjected to flotation. The top fraction was applied to a glow discharged carbon grid. Each sample was plunge-frozen in liquid ethane using an automated system and visualized on a Tecnai 20 electron microscope operating at a voltage of 200 kV.

## Data Availability Statement

The original contributions presented in the study are included in the article/Supplementary Material, further inquiries can be directed to the corresponding author.

## Author Contributions

JZ and JH designed the research. JZ performed the experiments. JZ and JH analyzed the data. JH wrote the manuscript.

## Conflict of Interest

The authors declare that the research was conducted in the absence of any commercial or financial relationships that could be construed as a potential conflict of interest.
