# Supplementary figures and images for: Self-Association of Purified Reconstituted ER Luminal Spacer Climp63
Source: Front Cell Dev Biol. 2020 Jun 16;8:500. doi: 10.3389/fcell.2020.00500 (PMC7308479; doi:10.3389/fcell.2020.00500)

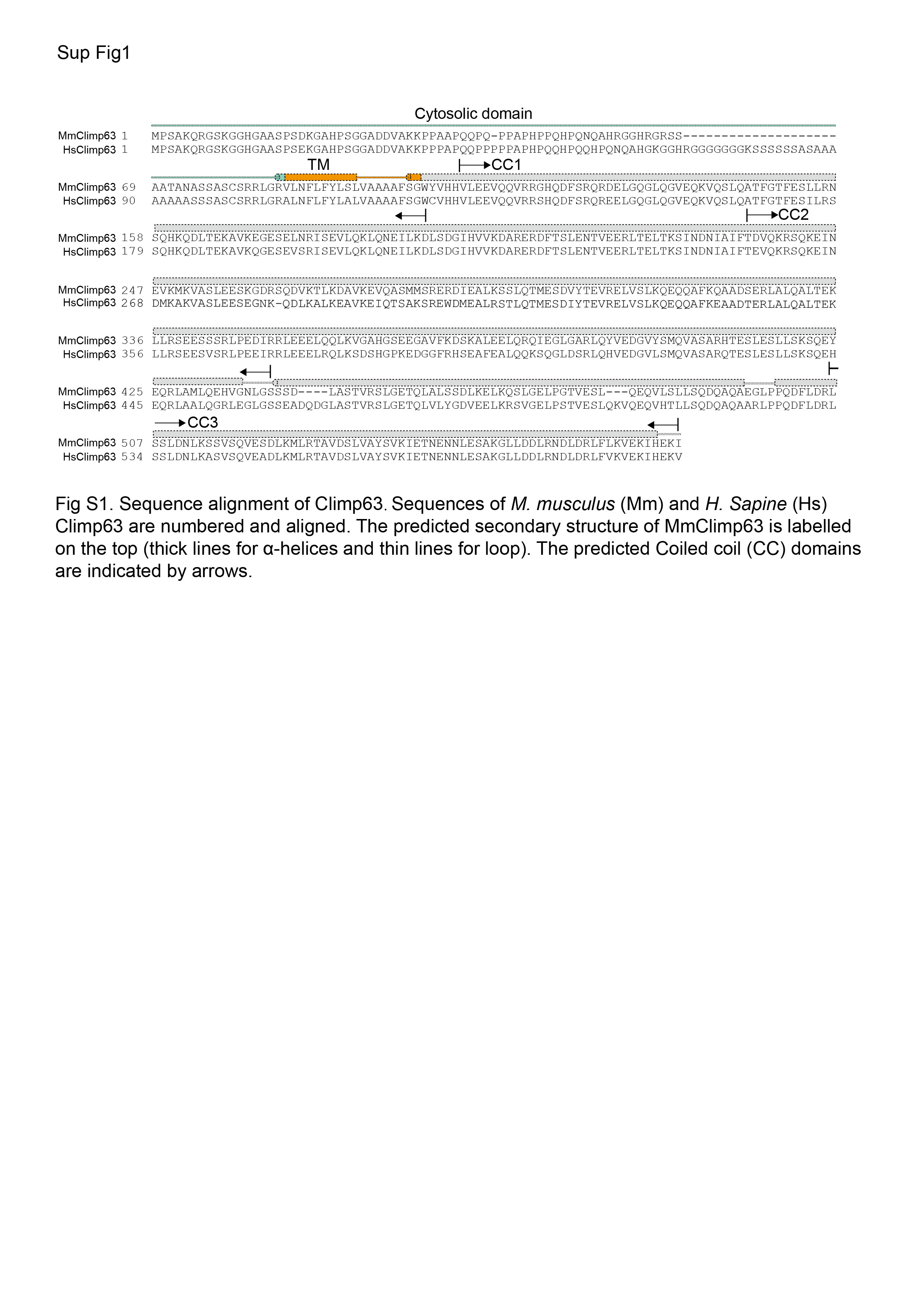

Supplement: Supplementary file 1 [file Image_1.jpg]

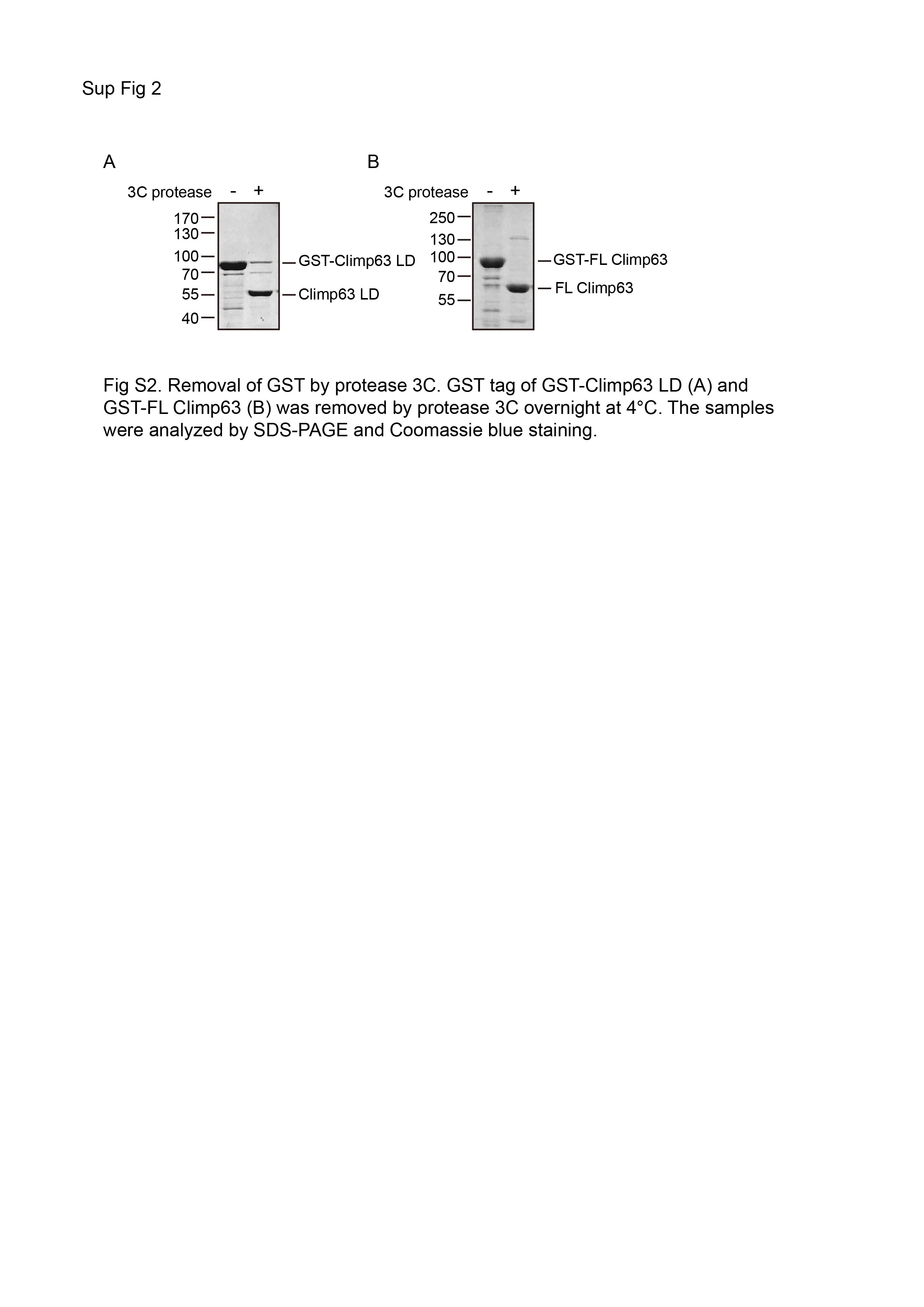

Supplement: Supplementary file 2 [file Image_2.jpg]

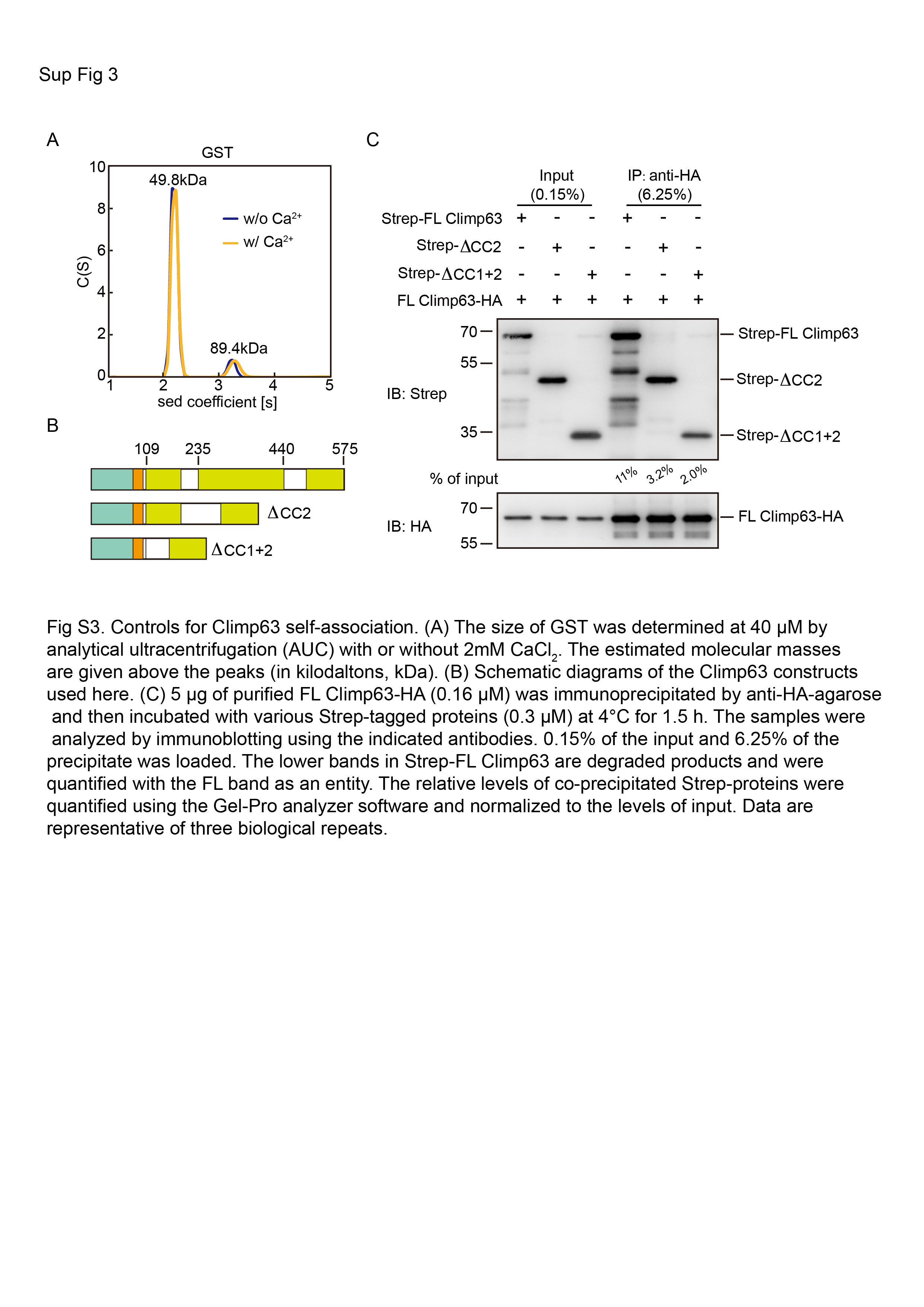

Supplement: Supplementary file 3 [file Image_3.jpg]

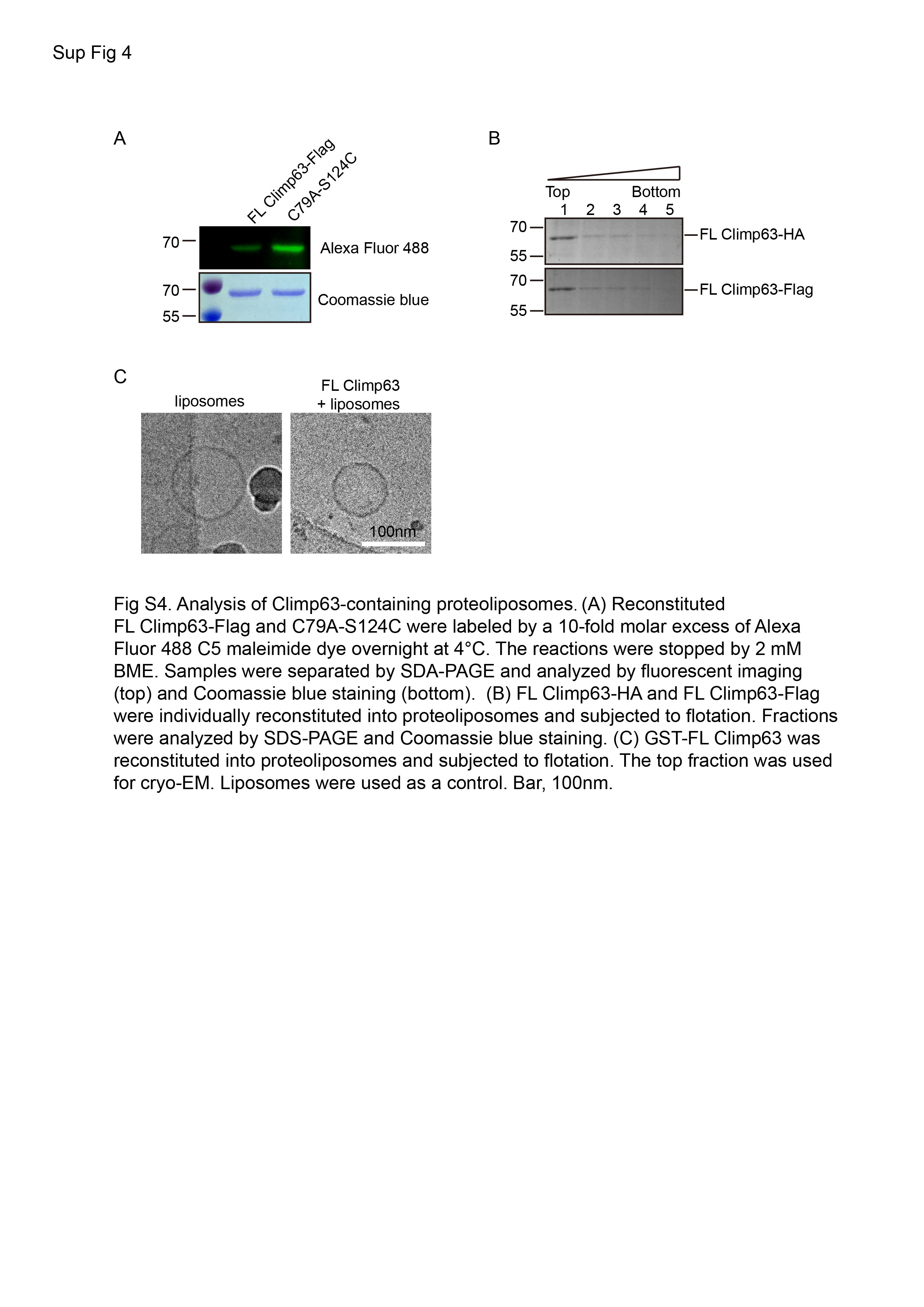

Supplement: Supplementary file 4 [file Image_4.jpg]
